# Trends in induction of labour and associated co-morbidities and demographics in Queensland, Australia from 2001 to 2020: a population-based study

**DOI:** 10.1186/s12884-025-07379-5

**Published:** 2025-03-26

**Authors:** Nigel Lee, Emma Ballard, Tracy Humphrey

**Affiliations:** 1 School of Nursing, Midwifery and Social Work, The University of Queensland, Level 3 Chamberlain Building, St Lucia, QLD 4072 Australia; 2 QIMR Berghofer Medical Research Institute, Herston, QLD Australia; 3 Clinical and Health Sciences, University of South Australia, Adelaide, South Australia Australia

**Keywords:** Vaginal birth, Induction of labour, Parity, Population trends

## Abstract

**Background:**

Amongst women who plan a vaginal birth at term, previous studies have reported that rates of induction of labour are increasing potentially impacting other labour and birth outcomes. Indications for induction of labour (IOL) have changed over time though the influences of parity and demographic factors such as age, ethnicity and regionality are not often considered. The aim of this study was to describe the changes in demographic, co-morbidity, IOL indication and clinical outcomes in women undertaking a planned cephalic vaginal birth at term over a 20 year period.

**Methods:**

A retrospective population-based study was undertaken using routinely collected anonymised perinatal data from Queensland, Australia from January 2001 to December 2020. We included all singleton term (≥ 37 weeks) planned vaginal births. A total of 836,065 births met the study criteria. Data for pregnancy complications and IOL indications were grouped by ICD-10 codes. Analysis was stratified by parity and presented as frequency and percentages over time and the difference in percentages between two defined years.

**Results:**

Rates of IOL increased by 15.5% (31.6 to 47.1%) in nulliparous and 14.6% (26.2 to 40.8% in multiparous women, most notable from 2015 onwards. Over the same period infants born between 37 and 38 weeks gestation increased by 13.9%. (18.1–32%). Amongst co-morbidities gestational diabetes increased from 3.8 to 12.8% and anaemia from 1.7 to 8.1%. As an indication for IOL prolonged pregnancy decreased from 41.0 to 11.2%. In nulliparous women the percentage of intact perineum decreased from 21.3 to 6.7% while episiotomy increased from 20.2 to 38.8%.

**Conclusions:**

We conclude that for women planning a vaginal birth not only has the rate of IOL increased substantially over the last two decades there also appears to be considerable interaction between demographic, co-morbidity, IOL indications and clinical outcomes that warrants further large population-based research.

**Supplementary Information:**

The online version contains supplementary material available at 10.1186/s12884-025-07379-5.

## Introduction

Planned cephalic vaginal birth at term, following either spontaneous onset or induced labour, is the safest and most cost effective mode of birth [1, 2]. Compared to elective caesarean section, planned vaginal birth is associated with a lower risk of short term complications such as infection and haemorrhage, and adverse outcomes in subsequent pregnancies [3]. While planned vaginal birth is undertaken by the majority of women and birthing persons, internationally the rates have decreased over time as elective caesarean section (CS) increases [4, 5]. Similarly rates of induction of labour (IOL) have also increased resulting in an overall decrease in spontaneous labour onset [6]. 

The decrease in spontaneous onset of labour as a percentage of planned vaginal birth may be due to changes in policy, clinical and societal factors over the years. A number of studies have cited changes in the main indications for IOL over time [7–9]. However these changes in indication are not consistent across studies or countries. An examination of IOL trends over 20 years in Iceland reported rises in IOL for gestational diabetes mellitus (GDM) and prolonged pregnancy, whereas a single site study from Australia reported a decrease in IOL for prolonged pregnancy and an increase for decreased fetal movements over a five year period [7, 8]. Both studies reported a rise in elective IOL, that is, IOL that does not have a clear obstetric or medical indication but may be influenced by other factors that would not otherwise exclude a spontaneous onset of labour. Shifting demographic factors such as maternal age, ethnicity and regionality may also be independent factors in changes in the distribution of IOL and spontaneous labour within planned vaginal birth over time [8, 10, 11]. 

Exploring trends in demographic characteristics and clinical indicators longitudinally can provide insights into the factors influencing variations in birth outcomes. While nulliparous and multiparous women are both exposed to co-morbidities and IOL there exists the potential for difference in outcomes. Differences between nulliparous and multiparous women’s experience of IOL have also been reported [12]. However, previous authors have noted that the reporting of labour and birth outcomes in population based studies of IOL are often not stratified by parity [13, 14]. This approach to amalgamate parity and other variables contributes to the difficulties in reporting trends, in birth outcomes for women who plan a vaginal birth at term with either a spontaneous or induced onset.

The aim of this study was to describe the changes in demographic, co-morbidity, IOL indication and present trends stratified by parity in clinical outcomes in women undertaking a planned cephalic vaginal birth at term over a 20 year period.

## Methods

A retrospective population-based study was undertaken using routinely collected perinatal data from Queensland, Australia from January 2001 to December 2020. Queensland is geographically the second largest State in Australia and accounts for approximately 20% of the Australian population. All Queensland hospitals (state and private) contribute perinatal data via the Perinatal Data Collection (PDC) form for each birth. The PDC records data on basic demographics, previous pregnancies and outcomes, pre-existing and pregnancy related co-morbidities, labour, birth, postnatal and neonatal outcomes. Common medical conditions and pregnancy complications are pre-defined on the form with free text boxes capturing any conditions not listed. Indications for IOL are entered as free text. This data is then coded to International Classification of Diseases, 10th revision (ICD-10) codes by the Statistical Services Branch (SSB) of the Queensland Health Department.

The anonymised dataset used for this study included all women with a singleton, cephalic presentation at term who planned a labour and vaginal birth with either spontaneous onset or IOL. Data for women with multiple pregnancies, preterm labour (< 37 weeks), planned caesarean section, malpresentation or stillbirth were not included. Some women would have recorded more than one pregnancy during the study period and therefore maybe represented more than once in the dataset.

Ethnicity was based on Australian Standard Classification of Countries for Social Statistics (ASCCSS) from January 2001 to June 2001 and from July 2001 onwards on the Standard Australian Classification of Countries (SACC). Country of Birth (CoB) was grouped into the five largest population groups in Australia; Australian, United Kingdom, India, China and New Zealand. All other CoB data was grouped as “Other”.

The PDC provides for a main and two sub indications for IOL, only the main indication was used in the study. Data for pregnancy complications and IOL indications were provided separately by the SSB with data as ICD 10 codes. We grouped data for all IOLs into the following categories: Prolonged pregnancy (41 weeks or more), diabetes (pre-existing and gestational), reduced fetal movements, Hypertension (including gestational, pre-eclampsia, eclampsia and HELLP), large for gestational age (LGA), small for gestational age (SGA), non-obstetric medical, obstetric medical, Labour complication, Fetal indication, advanced maternal age, psychosocial and elective. For outcomes such as LGA and SGA we are not able to define specific criteria as the ICD-10 codes provided are based on free text responses on the PDC form to a question regarding the main indication for induction. Therefore, the definition of LGA and SGA is open to the interpretation of the clinician initiating the induction or completing the form. The categorisation of specific ICD 10 codes in presented in Additional file 1. Data were reported in two calendar year periods from 1st January of the first year to 31st December of the second.

Data analysis was undertaken with Stata v14.1 (StatCorp, College Station, TX) and Microsoft Excel v2108. Categorical data is presented as frequency and percentage (%) for each two-year time period. Clinical outcomes are reported stratified by parity. For ease of interpretation, the difference in percentages between each specified two-year time point and 2001–2002 are presented for all variables in Additional file 2. Multivariable logistic regression models were used to examine IOL with the unadjusted model containing a fixed effect for year as a categorical variable and the adjusted model containing fixed effects for year, for private obstetrician, gestation, BMI 35 or over (All variables with reference category: No), country of birth (Reference category: Australia), maternal age (Reference category: 16 or younger) and regionality based on Australian Statistical Geography Standard Edition 3 (Reference category: major city). Residuals were examined. Predicted unadjusted and adjusted probabilities with 95% confidence intervals (CI) are plotted and presented in Additional file 3.

## Results

### Population demographics

Between January 2001 and December 2020, 836,065 births met the study criteria of live, term, singleton cephalic. Compared to 2001/2002 the number of teenage births more than halved from 7.3 to 3.3% in 2019/2020 and births to women over 35 years of age increased from 13.2 to 18.2%. The percentage of births occurring between 37 and 38 weeks gestation increased 13.9% over the study period while rates of post-term birth (41 weeks or more) decreased. Diversity of CoB increased during the study period with planned vaginal births to women from India increasing from 0.3% (2001/2002) to 4.4% (2019/2020). The majority of women received shared care through a General Practitioner (GP) or public hospital clinic with the percentage of women seeking antenatal care from a private obstetrician decreasing steadily over the 20 years (-12.6%) (Table 1; Additional file 2).

**Table 1:** Demographic characteristics of women planning a vaginal birth in Queensland 2001–2020

Patient characteristics	Total	2001–2002	2003–2004	2005–2006	2007–2008	2009–2010	2011–2012	2013–2014	2015–2016	2017–2018	2019–2020
	*N* = 836,065	*N* = 73,931	*N* = 73,680	*N* = 80,003	*N* = 87,002	*N* = 88,289	*N* = 89,101	*N* = 89,860	*N* = 87,644	*N* = 84,235	*N* = 82,320
**Maternal age**											
< 19	47,010 (5.6%)	5,397 (7.3%)	5,158 (7.0%)	5,212 (6.5%)	5,721 (6.6%)	5,689 (6.4%)	5,350 (6.0%)	4,812 (5.4%)	3,774 (4.3%)	3,142 (3.7%)	2,755 (3.3%)
20–29	406,939 (48.7%)	37,599 (50.9%)	36,031 (48.9%)	38,857 (48.6%)	42,599 (49.0%)	43,729 (49.5%)	44,352 (49.8%)	43,993 (49.0%)	42,135 (48.1%)	39,800 (47.2%)	37,844 (46.0%)
30–34	247,874 (29.6%)	21,181 (28.6%)	21,950 (29.8%)	23,589 (29.5%)	24,323 (28.0%)	24,211 (27.4%)	24,974 (28.0%)	26,806 (29.8%)	27,557 (31.4%)	26,555 (31.5%)	26,728 (32.5%)
35+	134,242 (16.1%)	9,754 (13.2%)	10,541 (14.3%)	12,345 (15.4%)	14,359 (16.5%)	14,660 (16.6%)	14,425 (16.2%)	14,249 (15.9%)	14,178 (16.2%)	14,738 (17.5%)	14,993 (18.2%)
**Gestation (weeks)**											
37–38	196,906 (23.6%)	13,416 (18.1%)	14,214 (19.3%)	15,648 (19.6%)	17,226 (19.8%)	18,326 (20.8%)	20,017 (22.5%)	21,776 (24.2%)	23,841 (27.2%)	26,105 (31.0%)	26,337 (32.0%)
39–40	501,001 (59.9%)	45,753 (61.9%)	45,892 (62.3%)	50,289 (62.9%)	53,916 (62.0%)	54,009 (61.2%)	53,500 (60.0%)	53,010 (59.0%)	50,592 (57.7%)	47,528 (56.4%)	46,512 (56.5%)
41+	138,158 (16.5%)	14,762 (20.0%)	13,574 (18.4%)	14,066 (17.6%)	15,860 (18.2%)	15,954 (18.1%)	15,584 (17.5%)	15,074 (16.8%)	13,211 (15.1%)	10,602 (12.6%)	9,471 (11.5%)
**Country of birth**											
Australia	644,879 (77.1%)	62,088 (84.0%)	61,212 (83.1%)	66,045 (82.6%)	70,317 (80.8%)	68,347 (77.4%)	67,570 (75.8%)	66,757 (74.3%)	63,842 (72.8%)	59,977 (71.2%)	58,724 (71.3%)
UK	20,180 (2.4%)	1,937 (2.6%)	1,824 (2.5%)	1,872 (2.3%)	2,106 (2.4%)	2,219 (2.5%)	2,130 (2.4%)	2,001 (2.2%)	2,107 (2.4%)	1,987 (2.4%)	1,997 (2.4%)
India	18,289 (2.2%)	258 (0.3%)	325 (0.4%)	391 (0.5%)	692 (0.8%)	1,703 (1.9%)	2,151 (2.4%)	2,605 (2.9%)	3,075 (3.5%)	3,491 (4.1%)	3,598 (4.4%)
China	12,201 (1.5%)	536 (0.7%)	471 (0.6%)	547 (0.7%)	731 (0.8%)	1,021 (1.2%)	1,440 (1.6%)	1,752 (1.9%)	1,940 (2.2%)	1,961 (2.3%)	1,802 (2.2%)
NZ	45,702 (5.5%)	3,174 (4.3%)	3,355 (4.6%)	3,825 (4.8%)	4,670 (5.4%)	5,320 (6.0%)	5,385 (6.0%)	5,444 (6.1%)	4,977 (5.7%)	4,953 (5.9%)	4,599 (5.6%)
Other	94,814 (11.3%)	5,938 (8.0%)	6,493 (8.8%)	7,323 (9.2%)	8,486 (9.8%)	9,679 (11.0%)	10,425 (11.7%)	11,301 (12.6%)	11,703 (13.4%)	11,866 (14.1%)	11,600 (14.1%)
**Region**											
Major city	547,004 (65.4%)	46,087 (62.3%)	46,905 (63.7%)	51,138 (63.9%)	55,970 (64.3%)	57,513 (65.1%)	58,194 (65.3%)	58,837 (65.5%)	58,741 (67.0%)	57,517 (68.3%)	56,102 (68.2%)
Inner regional	155,208 (18.6%)	14,263 (19.3%)	13,839 (18.8%)	15,251 (19.1%)	16,502 (19.0%)	16,515 (18.7%)	16,886 (19.0%)	16,887 (18.8%)	15,836 (18.1%)	14,652 (17.4%)	14,577 (17.7%)
Outer regional/ remote	133,853 (16.0%)	13,581 (18.4%)	12,936 (17.6%)	13,614 (17.0%)	14,530 (16.7%)	14,261 (16.2%)	14,021 (15.7%)	14,136 (15.7%)	13,067 (14.9%)	12,066 (14.3%)	11,641 (14.1%)
**Indigenous status** (*n* = 835,916)	52,775 (6.3%)	4,194 (5.7%)	4,253 (5.8%)	4,550 (5.7%)	4,960 (5.7%)	5,140 (5.8%)	5,584 (6.3%)	5,712 (6.4%)	6,018 (6.9%)	6,009 (7.1%)	6,355 (7.7%)
**Model of Care** (*n* = 833,656)											
Public hospital	338,205 (40.6%)	22,563 (30.6%)	24,801 (33.8%)	29,264 (36.7%)	41,371 (47.7%)	43,128 (49.0%)	40,156 (45.2%)	36,782 (41.1%)	34,290 (39.2%)	34,057 (40.5%)	31,793 (38.7%)
GP shared care	283,573 (34.0%)	28,379 (38.5%)	27,016 (36.8%)	28,313 (35.5%)	21,839 (25.2%)	21,630 (24.6%)	26,068 (29.3%)	30,355 (33.9%)	32,921 (37.7%)	32,403 (38.6%)	34,649 (42.2%)
Private Obstetrician	205,882 (24.7%)	22,373 (30.4%)	21,434 (29.2%)	22,106 (27.7%)	23,297 (26.9%)	23,047 (26.2%)	22,333 (25.1%)	21,494 (24.0%)	18,820 (21.5%)	16,377 (19.5%)	14,601 (17.8%)
Private Midwife	5,996 (0.7%)	345 (0.5%)	175 (0.2%)	106 (0.1%)	198 (0.2%)	258 (0.3%)	310 (0.3%)	951 (1.1%)	1,386 (1.6%)	1,202 (1.4%)	1,065 (1.3%)

Table is n (%)

A number of co-morbidities demonstrated substantial change over the study period. Most notable a diagnosis of diabetes increased from 3.8% in 2001/2002 to 12.8% in 2019/2020. Mental health diagnosis increased from 3.1 to 10.6% and anaemia from 2.3 to 8.1%. The increased rates for diabetes and anaemia were more prominent after 2015–2016. Prolonged pregnancy as a recorded co-morbidity decreased from 13.5 to 5.9% (Table 2).

**Table 2:** Trends in co-morbidities in planned vaginal birth in Queensland 2001–2020

	Total	2001–2002	2003–2004	2005- 2006	2007–2008	2009–2010	2011–2012	2013–2014	2015–2016	2017–2018	2019–2020
	*N* = 836,065	*N* = 73,931	*N* = 73,680	*N* = 80,003	*N* = 87,002	*N* = 88,289	*N* = 89,101	*N* = 89,860	*N* = 87,644	*N* = 84,235	*N* = 82,320
Hypertension	39,799 (4.8%)	4,913 (6.6%)	4,389 (6.0%)	4,148 (5.2%)	4,132 (4.7%)	3,787 (4.3%)	3,501 (3.9%)	3,355 (3.7%)	3,579 (4.1%)	4,078 (4.8%)	3,917 (4.8%)
Gestational diabetes	60,048 (7.2%)	2,774 (3.8%)	3,167 (4.3%)	3,644 (4.6%)	3,954 (4.5%)	4,381 (5.0%)	5,347 (6.0%)	6,648 (7.4%)	9,164 (10.5%)	10,436 (12.4%)	10,533 (12.8%)
Liver disease	5,042 (0.6%)	37 (0.1%)	87 (0.1%)	301 (0.4%)	404 (0.5%)	373 (0.4%)	456 (0.5%)	604 (0.7%)	1,013 (1.2%)	1,030 (1.2%)	737 (0.9%)
Term premature rupture of membranes	28,236 (3.4%)	1,813 (2.5%)	1,776 (2.4%)	2,053 (2.6%)	2,670 (3.1%)	3,084 (3.5%)	3,222 (3.6%)	3,455 (3.8%)	3,504 (4.0%)	3,326 (3.9%)	3,333 (4.0%)
Anaemia	26,268 (3.1%)	1,688 (2.3%)	1,362 (1.8%)	1,358 (1.7%)	1,670 (1.9%)	1,894 (2.1%)	1,723 (1.9%)	1,736 (1.9%)	2,569 (2.9%)	5,606 (6.7%)	6,662 (8.1%)
Mental health	39,000 (4.7%)	2,260 (3.1%)	1,770 (2.4%)	1,864 (2.3%)	2,307 (2.7%)	2,749 (3.1%)	3,183 (3.6%)	4,041 (4.5%)	5,221 (6.0%)	6,908 (8.2%)	8,697 (10.6%)
Antepartum haemorrhage	13,887 (1.7%)	1,612 (2.2%)	1,230 (1.7%)	1,173 (1.5%)	1,155 (1.3%)	1,368 (1.5%)	1,283 (1.4%)	1,389 (1.5%)	1,445 (1.6%)	1,628 (1.9%)	1,604 (1.9%)
Prolonged pregnancy	80,512 (9.6%)	10,010 (13.5%)	9,485 (12.9%)	10,150 (12.7%)	10,491 (12.1%)	8,946 (10.1%)	8,057 (9.0%)	7,086 (7.9%)	6,191 (7.1%)	5,248 (6.2%)	4,848 (5.9%)

Data is n (%)

### Changes in induction of labour

Substantial changes in a number of indications for IOL across the study period are illustrated in Table 3. A number of these reflect the changes in co-morbidities. The rate of IOL for prolonged pregnancy fell 29.4% from 40.6% in 2001/2002 to 11.2% in 2019/2020. Rates for Hypertension IOL also fell 5.4%. In contrary rates of IOL for Diabetes increased 10.9%, Reduced fetal movements increased 8.6% and LGA by 7.7%. Changes appeared to accelerate from 2015 to 2016 onwards for each of these variables. While accounting for a relatively small proportion of IOL, rates for advanced maternal age demonstrated the largest percentage increase across the study period from 0.6 to 3.6% and doubling from 1.8% in 2015–2016. Rates of elective IOL peaked at 20.7% in 2011–2012 then decreased to 15.2% in 2019–2021. However, in 2019/2020 elective IOL is proportionally the most common indication for IOL (15.2%) followed by diabetes (14.4%) (Table 3; Additional file 2).

**Table 3:** Trends in indications for induction of labour 2001–2020

	Total	2001–2002	2003–2004	2005–2006	2007–2008	2009–2010	2011–2012	2013–2014	2015–2016	2017–2018	2019–2020
	*N* = 276,501	*N* = 23,230	*N* = 22,494	*N* = 23,910	*N* = 25,259	*N* = 25,212	*N* = 26,434	*N* = 28,132	*N* = 30,967	*N* = 34,912	*N* = 35,951
Prolonged pregnancy	74,509 (27.0%)	9,422 (40.6%)	9,213 (41.0%)	9,798 (41.0%)	10,071 (39.9%)	8,449 (33.5%)	7,157 (27.1%)	6,501 (23.1%)	5,468 (17.7%)	4,392 (12.6%)	4,038 (11.2%)
Diabetes	25,928 (9.4%)	806 (3.5%)	870 (3.9%)	1,208 (5.1%)	1,628 (6.4%)	1,743 (6.9%)	2,248 (8.5%)	2,952 (10.5%)	4,205 (13.6%)	5,084 (14.6%)	5,184 (14.4%)
Reduced fetal movements	10,418 (3.8%)	299 (1.3%)	317 (1.4%)	315 (1.3%)	232 (0.9%)	196 (0.8%)	377 (1.4%)	668 (2.4%)	1,298 (4.2%)	3,149 (9.0%)	3,567 (9.9%)
Hypertension	26,183 (9.5%)	2,931 (12.6%)	2,709 (12.0%)	2,576 (10.8%)	2,578 (10.2%)	2,480 (9.8%)	2,510 (9.5%)	2,504 (8.9%)	2,606 (8.4%)	2,712 (7.8%)	2,577 (7.2%)
TPROM	28,738 (10.4%)	2,690 (11.6%)	2,577 (11.5%)	2,522 (10.5%)	2,979 (11.8%)	3,067 (12.2%)	3,016 (11.4%)	3,126 (11.1%)	3,057 (9.9%)	2,914 (8.3%)	2,790 (7.8%)
Large for gestational age	11,456 (4.1%)	418 (1.8%)	415 (1.8%)	488 (2.0%)	541 (2.1%)	532 (2.1%)	674 (2.5%)	789 (2.8%)	1,458 (4.7%)	2,730 (7.8%)	3,411 (9.5%)
Small for gestational age	13,911 (5.0%)	913 (3.9%)	831 (3.7%)	847 (3.5%)	814 (3.2%)	997 (4.0%)	1,174 (4.4%)	1,459 (5.2%)	1,985 (6.4%)	2,467 (7.1%)	2,424 (6.7%)
Non obstetric medical	7,449 (2.7%)	562 (2.4%)	510 (2.3%)	544 (2.3%)	600 (2.4%)	764 (3.0%)	622 (2.4%)	746 (2.7%)	793 (2.6%)	1,140 (3.3%)	1,168 (3.2%)
Obstetric medical	14,435 (5.2%)	1,030 (4.4%)	1,070 (4.8%)	1,230 (5.1%)	1,298 (5.1%)	1,208 (4.8%)	1,420 (5.4%)	1,567 (5.6%)	1,807 (5.8%)	1,928 (5.5%)	1,877 (5.2%)
Labour complication	6,168 (2.2%)	650 (2.8%)	345 (1.5%)	307 (1.3%)	355 (1.4%)	781 (3.1%)	542 (2.1%)	637 (2.3%)	770 (2.5%)	912 (2.6%)	869 (2.4%)
Fetal indication	8,775 (3.2%)	567 (2.4%)	440 (2.0%)	500 (2.1%)	571 (2.3%)	664 (2.6%)	885 (3.3%)	1,151 (4.1%)	1,292 (4.2%)	1,337 (3.8%)	1,368 (3.8%)
Advanced maternal age	5,103 (1.8%)	148 (0.6%)	110 (0.5%)	142 (0.6%)	165 (0.7%)	188 (0.7%)	218 (0.8%)	509 (1.8%)	976 (3.2%)	1,335 (3.8%)	1,312 (3.6%)
Elective IOL	42,829 (15.5%)	2,786 (12.0%)	3,052 (13.6%)	3,408 (14.3%)	3,332 (13.2%)	4,042 (16.0%)	5,483 (20.7%)	5,382 (19.1%)	5,114 (16.5%)	4,782 (13.7%)	5,448 (15.2%)
Psychosocial	648 (0.2%)	56 (0.2%)	57 (0.3%)	70 (0.3%)	90 (0.4%)	81 (0.3%)	73 (0.3%)	64 (0.2%)	29 (0.1%)	58 (0.2%)	70 (0.2%)
	36,259 (7.7%)	3,603 (8.7%)	3,604 (8.6%)	3,755 (8.2%)	4,142 (8.4%)	4,192 (8.5%)	4,187 (8.5%)	3,867 (7.8%)	3,440 (7.0%)	2,850 (6.0%)	2,619 (5.8%)

Data is n (%)

### Changes in birth outcomes by parity

For both nulliparous and multiparous women, the percentage of IOL increased from 31.4% in 2001/2002 to 43.6% in 2019/2020. Over the same period the use of epidural or spinal analgesia increased from 25.7 to 41.1%. Unassisted vaginal birth decreased from 77.8 to 70.5% with assisted births increasing from 9.9% in 2001/2002 to 13.3% in 2013/2014 with a reduced increment to 13.9% in 2019/2020. A similar trend is noted in the percentage of CS which increased from 9.0% in 2001/2002 to 14.4% in 2007/2008 and plateaued (14.0 − 14.7%) until 2019/2020 where the rate increased to 15.5%.

The rate of IOL In nulliparous women increased by 15.5% (31.6 to 47.1%) and 14.6% (26.2 to 40.8%). in multiparous women In both groups the rate of increase was most notable from 2015 to 2016 onwards (Tables 4 and 5, Additional file 2). Following regression analysis the adjusted predicted probability values with 95% confidence intervals for IOL and yearly pattern were similar to those seen for the unadjusted model (Additional file 3). The number of nulliparous women utilising epidural or spinal analgesia during labour increased 18% from 39.3% in 2001–2002 to 57.3% in 2019–2020. The use of regional analgesia for multiparous women also increased 12.8% over the study period (Table 4, Additional file 2).

**Table 4:** Trends in clinical outcomes for nulliparous planned vaginal births 2001–2020

	Total	2001–2002	2003–2004	2005–2006	2007–2008	2009–2010	2011–2012	2013–2014	2015–2016	2017–2018	2019–2020
	*N* = 367,894	*N* = 32,462	*N* = 31,773	*N* = 34,187	*N* = 37,564	*N* = 38,917	*N* = 39,703	*N* = 40,326	*N* = 38,766	*N* = 37,111	*N* = 37,085
IOL	134,156 (36.5%)	11,078 (34.1%)	10,645 (33.5%)	11,351 (33.2%)	12,201 (32.5%)	12,291 (31.6%)	13,116 (33.0%)	14,108 (35.0%)	15,156 (39.1%)	16,736 (45.1%)	17,474 (47.1%)
Spontaneous onset of labour	233,738 (63.5%)	21,384 (65.9%)	21,128 (66.5%)	22,836 (66.8%)	25,363 (67.5%)	26,626 (68.4%)	26,587 (67.0%)	26,218 (65.0%)	23,610 (60.9%)	20,375 (54.9%)	19,611 (52.9%)
Augmentation (*n* = 233,738 )	119,790 (51.2%)	11,372 (53.2%)	11,383 (53.9%)	11,880 (52.0%)	13,302 (52.4%)	13,356 (50.2%)	12,983 (48.8%)	12,893 (49.2%)	12,144 (51.4%)	10,678 (52.4%)	9,799 (50.0%)
Epidural analgesia	171,602 (46.6%)	12,770 (39.3%)	13,056 (41.1%)	14,471 (42.3%)	16,214 (43.2%)	17,012 (43.7%)	18,040 (45.4%)	19,213 (47.6%)	19,376 (50.0%)	20,207 (54.5%)	21,243 (57.3%)
Mode of birth											
Unassisted vaginal birth	205,203 (55.8%)	20,286 (62.5%)	19,152 (60.3%)	19,963 (58.4%)	21,056 (56.1%)	21,635 (55.6%)	21,713 (54.7%)	22,006 (54.6%)	20,884 (53.9%)	19,263 (51.9%)	19,245 (51.9%)
Assisted vaginal birth	81,447 (22.1%)	5,800 (17.9%)	5,939 (18.7%)	6,552 (19.2%)	7,890 (21.0%)	8,715 (22.4%)	9,071 (22.8%)	9,599 (23.8%)	9,568 (24.7%)	9,233 (24.9%)	9,080 (24.5%)
Caesarean section	81,244 (22.1%)	6,376 (19.6%)	6,682 (21.0%)	7,672 (22.4%)	8,618 (22.9%)	8,567 (22.0%)	8,919 (22.5%)	8,721 (21.6%)	8,314 (21.4%)	8,615 (23.2%)	8,760 (23.6%)
Perineal status (*n* = 286,526)											
Intact	37,338 (13.0%)	5,562 (21.3%)	5,130 (20.4%)	5,195 (19.6%)	4,521 (15.6%)	3,730 (12.3%)	3,678 (12.0%)	3,212 (10.2%)	2,465 (8.1%)	1,942 (6.8%)	1,903 (6.7%)
1st degree	68,635 (24.0%)	6,824 (26.2%)	6,230 (24.8%)	6,545 (24.7%)	7,647 (26.4%)	8,401 (27.7%)	8,008 (26.0%)	7,406 (23.4%)	6,679 (21.9%)	5,672 (19.9%)	5,223 (18.5%)
2nd degree	90,533 (31.6%)	7,503 (28.8%)	7,083 (28.2%)	7,783 (29.4%)	9,006 (31.1%)	9,889 (32.6%)	10,029 (32.6%)	10,566 (33.4%)	10,229 (33.6%)	9,215 (32.4%)	9,230 (32.6%)
3rd /4th degree	11,634 (4.1%)	933 (3.6%)	909 (3.6%)	1,011 (3.8%)	1,285 (4.4%)	1,353 (4.5%)	1,469 (4.8%)	1,422 (4.5%)	1,212 (4.0%)	1,064 (3.7%)	976 (3.4%)
Episiotomy	78,386 (27.4%)	5,264 (20.2%)	5,739 (22.9%)	5,981 (22.6%)	6,485 (22.4%)	6,959 (22.9%)	7,572 (24.6%)	8,989 (28.5%)	9,845 (32.4%)	10,576 (37.1%)	10,976 (38.8%)
Infant birth weight > 4200 g	18,202 (4.9%)	1,879 (5.8%)	1,751 (5.5%)	1,905 (5.6%)	2,139 (5.7%)	2,083 (5.4%)	2,165 (5.5%)	1,971 (4.9%)	1,633 (4.2%)	1,303 (3.5%)	1,373 (3.7%)

Data is n (%)

**Table 5:** Trends in clinical outcomes for multiparous planned vaginal births 2001–2020

	Total	2001–2002	2003–2004	2005–2006	2007–2008	2009–2010	2011–2012	2013–2014	2015–2016	2017–2018	2019–2020
	*N* = 468,171	*N* = 41,469	*N* = 41,907	*N* = 45,816	*N* = 49,438	*N* = 49,372	*N* = 49,398	*N* = 49,534	*N* = 48,878	*N* = 47,124	*N* = 45,235
IOL	142,345 (30.4%)	12,152 (29.3%)	11,849 (28.3%)	12,559 (27.4%)	13,058 (26.4%)	12,921 (26.2%)	13,318 (27.0%)	14,024 (28.3%)	15,811 (32.3%)	18,176 (38.6%)	18,477 (40.8%)
Spontaneous onset of labour	325,826 (69.6%)	29,317 (70.7%)	30,058 (71.7%)	33,257 (72.6%)	36,380 (73.6%)	36,451 (73.8%)	36,080 (73.0%)	35,510 (71.7%)	33,067 (67.7%)	28,948 (61.4%)	26,758 (59.2%)
Augmentation (*n* = 325,826)	103,907 (31.9%)	11,704 (39.9%)	11,601 (38.6%)	12,061 (36.3%)	12,816 (35.2%)	11,088 (30.4%)	9,956 (27.6%)	9,714 (27.4%)	9,483 (28.7%)	8,388 (29.0%)	7,096 (26.5%)
Epidural analgesia	91,892 (19.6%)	6,267 (15.1%)	6,788 (16.2%)	7,462 (16.3%)	8,190 (16.6%)	8,718 (17.7%)	9,147 (18.5%)	9,914 (20.0%)	10,940 (22.4%)	11,860 (25.2%)	12,606 (27.9%)
Mode of birth											
Unassisted vaginal birth	410,691 (87.7%)	37,285 (89.9%)	37,328 (89.1%)	40,732 (88.9%)	43,405 (87.8%)	43,175 (87.4%)	43,180 (87.4%)	43,313 (87.4%)	42,471 (86.9%)	40,946 (86.9%)	38,856 (85.9%)
Assisted vaginal birth	21,083 (4.5%)	1,530 (3.7%)	1,571 (3.7%)	1,731 (3.8%)	2,072 (4.2%)	2,211 (4.5%)	2,433 (4.9%)	2,366 (4.8%)	2,416 (4.9%)	2,373 (5.0%)	2,380 (5.3%)
Caesarean section	36,397 (7.8%)	2,654 (6.4%)	3,008 (7.2%)	3,353 (7.3%)	3,961 (8.0%)	3,986 (8.1%)	3,785 (7.7%)	3,855 (7.8%)	3,991 (8.2%)	3,805 (8.1%)	3,999 (8.8%)
Perineal status (*n* = 431,645)											
Intact	175,733 (40.7%)	18,657 (48.1%)	18,669 (48.0%)	20,714 (48.8%)	19,520 (42.9%)	17,687 (39.0%)	17,857 (39.2%)	18,108 (39.7%)	16,442 (36.6%)	14,820 (34.2%)	13,259 (32.2%)
1st degree	128,126 (29.7%)	10,806 (27.8%)	10,738 (27.6%)	11,458 (27.0%)	14,060 (30.9%)	14,920 (32.9%)	14,381 (31.6%)	13,088 (28.7%)	13,059 (29.1%)	13,155 (30.4%)	12,461 (30.2%)
2nd degree	98,895 (22.9%)	6,850 (17.6%)	6,959 (17.9%)	7,875 (18.5%)	9,287 (20.4%)	10,059 (22.2%)	10,544 (23.1%)	11,416 (25.0%)	12,030 (26.8%)	11,908 (27.5%)	11,967 (29.0%)
3rd /4th degree	4,081 (0.9%)	272 (0.7%)	268 (0.7%)	253 (0.6%)	381 (0.8%)	416 (0.9%)	493 (1.1%)	499 (1.1%)	547 (1.2%)	490 (1.1%)	462 (1.1%)
Episiotomy	24,810 (5.7%)	2,229 (5.7%)	2,264 (5.8%)	2,162 (5.1%)	2,227 (4.9%)	2,289 (5.0%)	2,306 (5.1%)	2,548 (5.6%)	2,791 (6.2%)	2,929 (6.8%)	3,065 (7.4%)
Infant birth weight > 4200 g	36,259 (7.7%)	3,603 (8.7%)	3,604 (8.6%)	3,755 (8.2%)	4,142 (8.4%)	4,192 (8.5%)	4,187 (8.5%)	3,867 (7.8%)	3,440 (7.0%)	2,850 (6.0%)	2,619 (5.8%)

Data is n (%)

The percentage of unassisted vaginal birth in nulliparous women decreased 10.6–51.9%. This resulted in a 6.6% increase in assisted births and 4.0% increase in CS. In multiparous women unassisted vaginal birth also decreased 4.0% with an increase of 2.4% in CS and 1.6% in assisted birth (Tables 4 and 5, Additional file 2).

The rate of intact perineum in nulliparous women fell 14.6% from 21.3% in 2001–2002 to 6.7% in 2019–2020 while over the same period episiotomy use increased 18.6% from 20.2 to 38.8%. Severe perineal trauma (3rd and 4th degree) peaked at 4.8% in 2011–2012 before declining by 2019–2021 to the same level as 2001–2002 (3.4%) (Table 4; Additional file 2). A similar trend was noted in multiparous women where intact perineum rates fell 15.9%. This was largely reflected in increases in 2nd degree injury of 11.4% and episiotomy (1.7%). The incidence of severe perineal injury was stable over the study period and largely unchanged since 2011–2012 (1.1 − 1.2%) (Table 5; Additional file 2).

## Discussion

This retrospective study illustrated a number of demographic and clinical changes in the population of women planning a vaginal birth over a 20 year period. Notably our study found substantial increases in IOL in both nulliparous and multiparous women. The number of babies born between 37 and 38 weeks gestation also increased. There were also substantial changes to the rates of IOL for particular indications such as diabetes and prolonged labour.

Changes in ethnic diversity reflect those in the Australia population more broadly, specifically the growth the Indian diaspora [15]. Whilst variations in indications for IOL may be more reflective of changes in clinical management of co-morbidities such as diabetes [16] and clinician attitudes to IOL in response to more recent research [17]. However a notable finding of the study is that for a number of IOL and clinical variables the rate of change appears to accelerate from the 2013/14–2015/2016 period. This phenomenon was also noted in a 20 year trend analysis of IOL in Iceland, though the data in this study was reported in five to six year intervals [8]. 

Prolonged pregnancy demonstrated a substantial reduction in both reported co-morbidity and indication of IOL over the study period. This was also noted in previous studies of a similar population, however that data was limited to 2015 to 2020 [7]. It is contrary to the trend data from Iceland that noted an increase in IOL for prolonged pregnancy [8]. The publication of RCTs supporting IOL to avoid complications of prolonged pregnancy [17–19] and the subsequent noted change in practice in some studies towards increased IOL rates at 39 weeks [20] may be a contributing factor to the noted decline in prolonged pregnancy. This would only be the case if IOL occurred prior to 41 weeks to avoid reaching that gestation and the indication was coded to another co-morbidity. It is noteworthy that in the trend data the decline in IOL for prolonged pregnancy appears to accelerate in the 2009–2010 period (-6.4%) with similar percentage reduction up until 2017–2018. This would seem to precede any significant change in research, practice or clinician attitude towards prolonged pregnancy. The period 2009–2010 to 2017–2018 does coincide with an accelerated increase in the rates of IOL for diabetes. It may be that the reduction in IOL numbers for prolonged pregnancy are related to increased earlier induction for other co-morbidities such as diabetes.

IOL for diabetes increased over four-fold during the study period despite gestational diabetes not being a specific or routine indication for IOL in either the Queensland or international guidelines [21]. While a number of authors have attributed some causation to this increase to changes in diagnostic thresholds ratified in Australia in 2014 [22, 23] other studies have found little or no influence [24] or highlight changes in demographics such as ethnicity and regionality as contributing factors [22]. Our study data indicates that the Indian diaspora population recorded the largest increase of all main Australian population groups. Women of South Asian ethnic groups, inclusive of India, are at a higher risk of diabetes independent of other risk factors such as raised BMI [24, 25]. Other studies have suggested that older age and residing in regional and remote areas also contributes to a risk in diabetes. In this study the pecentage of women in the 35 years or older age group did increase over time, though residency in a regional or remote area decreased after the 2025 − 2016 period. This suggests that the interaction between demographic influences on IOL is likely to be quite complex.

Clinician concern over risk of macrosomia often associated with diabetes in pregnancy may also be influential on the doubling of the rate of IOL for LGA seen in the dataset. Again, this occurred predominantly after the 2015–2016 time period. While most guidelines do not recommend IOL for macrosomia explicitly, the clinical guidelines for the State in which the study population resides does, based on weight at gestation criteria. Specifically 3500 g at 36 weeks, 3700 at 37 weeks and 3800 g at 38 weeks [26]. However such an approach relies on estimation of fetal weight by ultrasound and the potential for significant variations in this mode of assessment has been highlighted in past and more recent studies [27, 28]. The increase in IOL for both diabetes and LGA may be impacting on changes in two other variables, gestation at birth and infant weight, The percentage of infants born between 37 and 38 weeks increased noticeably after 2015–2016 period, as the number of infants born with a weight greater than 4,200 g decreased. Studies exploring the effect of early term induction (38 weeks) on childhood development have reported lower school performance with planned vaginal, either spontaneous or induced, birth at 40 weeks onwards [29]. An objective of glycaemic control and IOL for suspected LGA is to reduce the risk of LGA and subsequent complications such as shoulder dystocia. Only one randomised trial of IOL versus expectant management demonstrated a statistically significant reduction in shoulder dystocia. Observational studies have not reported an association between early IOL and a reduction in shoulder dystocia though did report an increase in CS associated with IOL [30, 31]. Other longitudinal studies of IOL for LGA also demonstrated significantly reduced birthweight in the IOL cohorts compared to expectant management associated with increased hospitalisations and special needs to age 8 years [32]. 

Across the 20 year study period the rate of intrapartum CS remained relatively stable in both nulliparous and multiparous women. This suggests that the increase in CS reported in both Queensland and national perinatal data are related predominantly to non-labour CS [6]. However study data indicated that more than one in five nulliparous women with a term singleton cephalic pregnancy and planning vaginal birth experienced a CS. Previous studies have suggested that IOL, epidural/spinal use and augmentation increase the risk of CS in low-risk nulliparous women [33, 34]. In our dataset, while the rate of augmentation in nulliparous women was 50% this remained stable over the study period though rates of IOL and epidural rates increased. The ARRIVE trial reported a lower CS rate with IOL compared to spontaneous onset though critiques of the research have cited the large number of women who declined to participate as a potential for selection bias raising questions regarding overall generalisability [17, 35]. Previous retrospective studies of data from a similar population to our study have reported that the CS rate was higher in the IOL group compared to spontaneous onset for women planning a vaginal birth [36]. 

An interesting outcome in our data was the substantial reduction in intact perinium and to a lesser extent first degree injury, in nulliparous women across the time period. This was concurrent with a similar increase in the rate of episiotomy. Over the study period there was some variation in the rate of severe perineal injury though this was not linear with the increase in episiotomy. The potential for episiotomy to be protective of severe perineal trauma in nulliparous remains contentious with recent RCTs and reviews questioning any benefit with the possible exception of assisted birth [37–39]. However the rise in episiotomy in nulliparous women was greater than the percent increase in assisted vaginal births. Recent rises in episiotomy use in Australia have been attributed to increased implementation of perineal protection bundles however associated reductions in severe perineal trauma have not been demonstrated in unassisted vaginal births [40]. 

A strength of our study is the access to a large dataset including all births over a 20 year period that met the study criteria for planned vaginal births. The data is validated by the Queensland Health Department SSB [41] prior to release which contributes to the high quality of the dataset with less than 0.03% missing data. Limitations of the study arise from the descriptive retrospective design that cannot demonstrate causation or association. The ICD-10 coding of the data relies on the responses from clinicians which may vary in accuracy and definitions applied to some variables. For example, it was evident in a very small number of cases when comparing codes for prolonged pregnancy to stated gestation at birth that, this did not always align suggesting some individual interpretation of the definition of prolonged pregnancy. Similarly, the potential for varying interpretations of free text entries, particularly those relating to IOL indications, may still occur.

## Conclusion

This study has illustrated the demographics and clinical changes in a large population of women planning a vaginal birth at term over a 20 year period. Our findings have mapped changes in the birthing population such as age, country of birth and gestation at onset of labour along with substantial changes in the main indications for IOL including a collapse in the rates for prolonged pregnancy and a surge in IOL for diabetes. We conclude that for women planning a vaginal birth not only has the rate of IOL increased substantially over the last two decades there also appears to be considerable change in demographic, co-morbidity, IOL indications and clinical outcomes that warrants further large population-based research at their interaction.

## Electronic supplementary material

Below is the link to the electronic supplementary material.

Supplementary Material 1

Supplementary Material 2

Supplementary Material 3

## Data Availability

The datasets generated for the current study are not publicly available due access restrictions required by the Queensland Perinatal Dataset Data Custodians. Analysed data are available from the corresponding author on reasonable request.

## Abbreviations

ASCCSS: Australian Standard Classification of Countries for Social Statistics
CoB: Country of Birth
CS: Caesarean section
HELLP: Haemolysis, Elevated Liver enzymes and Low Platelets
ICD-10: International Classification of Diseases, Tenth Revision
IOL: Induction of Labour
LGA: Large for Gestational Age
PDC: Perinatal Data Collection
SAC: Standard Australian Classification of Countries
SGA: Large for Gestational Age
SSB: Statistical Services Branch
