# Supplementary material for: Trends in induction of labour and associated co-morbidities and demographics in Queensland, Australia from 2001 to 2020: a population-based study
Source: BMC Pregnancy Childbirth. 2025 Mar 26;25:354. doi: 10.1186/s12884-025-07379-5 (PMC11938751; doi:10.1186/s12884-025-07379-5)
Supplement: Supplementary file 1 — Supplementary Material 1 [file 12884_2025_7379_MOESM1_ESM.pdf]

Table 1: Categorisation of ICD-10 codes (12th Edition) of main indication for induction of labour for all women

ICD 10 code	ICD 10 category	Induction of labour indication category
A (all codes)	Infectious and parasitic diseases	Non obstetric medical
B (all codes)	Infectious and parasitic diseases	Non obstetric medical
C (all codes)	Neoplastic	Non obstetric medical
D (all codes)	Neoplastic	Non obstetric medical
E (all codes)	Endocrine, nutritional and metabolic diseases	Non obstetric medical
E6613	Endocrine, nutritional and metabolic diseases	Obsteric medical
E6623	Endocrine, nutritional and metabolic diseases	Obsteric medical
E668	Endocrine, nutritional and metabolic diseases	Obsteric medical
E669	Endocrine, nutritional and metabolic diseases	Obsteric medical
E6690	Endocrine, nutritional and metabolic diseases	Obsteric medical
E6691	Endocrine, nutritional and metabolic diseases	Obsteric medical
E6692	Endocrine, nutritional and metabolic diseases	Obsteric medical
E6693	Endocrine, nutritional and metabolic diseases	Obsteric medical
F (all codes)	Mental and behavioural disorders	Non obstetric medical
G (all codes)	Diseases of the nervous system	Non obstetric medical
H (all codes)	Diseases of the eye and adnexa and ear	Non obstetric medical
I (all codes)	Diseases of the circulatory system	Non obstetric medical
J (all codes)	Diseases of the respiratory system	Non obstetric medical
K (all codes)	Diseases of the digestive system	Non obstetric medical
L (all codes)	Diseases of the skin and subcutaneous tissue	Non obstetric medical
M (all codes)	Diseases of the musculoskeletal system and connective tissue	Non obstetric medical
N (all codes)	Diseases of the genitourinary system	Non obstetric medical
O001	Tubal pregnancy	Obsteric medical
O028	Other specified abnormal products of conception	Obsteric medical
O048	Medical abortion, complete or unspecified, with other and unspecified complications	Obsteric medical
O077	Other and unspecified failed attempted abortion, complicated by embolism	Obsteric medical
O10	Pre-existing hypertension in pregnancy, childbirth and the puerperium	Hypertensive disorder (include PE & Eclampsia)

O100	Pre-existing essential hypertension complicating pregnancy, childbirth and the puerperium	Hypertensive disorder (include PE & Eclampsia)
O102	Pre-existing hypertensive kidney disease complicating pregnancy, childbirth and the puerperium	Hypertensive disorder (include PE & Eclampsia)
O104	Pre-existing secondary hypertension complicating pregnancy, childbirth and the puerperium	Hypertensive disorder (include PE & Eclampsia)
O11	Pre-eclampsia superimposed on chronic hypertension	Hypertensive disorder (include PE & Eclampsia)
O120	Gestational oedema	Obsteric medical
O121	Gestational proteinuria	Obsteric medical
O122	Gestational oedema with proteinuria	Obsteric medical
O13	Gestational [pregnancy-induced] hypertension	Hypertensive disorder (include PE & Eclampsia)
O140	Mild to moderate pre-eclampsia	Hypertensive disorder (include PE & Eclampsia)
O141	Severe pre-eclampsia	Hypertensive disorder (include PE & Eclampsia)
O142	HELLP syndrome	Hypertensive disorder (include PE & Eclampsia)
O149	Pre-eclampsia, unspecified	Hypertensive disorder (include PE & Eclampsia)
O150	Eclampsia in pregnancy	Hypertensive disorder (include PE & Eclampsia)
O151	Eclampsia in labour	Hypertensive disorder (include PE & Eclampsia)
O159	Eclampsia unspecified as to time period	Hypertensive disorder (include PE & Eclampsia)
O16	Eclampsia in labour	Hypertensive disorder (include PE & Eclampsia)
O200	Threatened abortion	Obsteric medical
O208	Other haemorrhage in early pregnancy	Obsteric medical
O209	Haemorrhage in early pregnancy, unspecified	Obsteric medical
O210	Hyperemesis gravidarum	Obsteric medical

O211	Hyperemesis gravidarum with metabolic disturbance	Obsteric medical
O212	Vomiting in late pregnancy	Obsteric medical
O218	Other vomiting complicating pregnancy	Obsteric medical
O219	Vomiting in pregnancy, not elsewhere classified	Obsteric medical
O220	Varicose veins of lower extremity in pregnancy	Obsteric medical
O221	Genital varices in pregnancy	Obsteric medical
O222	Superficial thrombophlebitis in pregnancy	Obsteric medical
O223	Deep phlebothrombosis in pregnancy	Obsteric medical
O224	Haemorrhoids in pregnancy	Obsteric medical
O229	Venous condition in pregnancy	Obsteric medical
O230	Infections of kidney in pregnancy	Obsteric medical
O233	Infections of other parts of urinary tract in pregnancy	Obsteric medical
O234	Unspecified infection of urinary tract in pregnancy	Obsteric medical
O235	Infections of the genital tract in pregnancy	Obsteric medical
O240	Pre-existing Type 1 diabetes mellitus in pregnancy, childbirth and the puerperium	Diabetes
O241	Pre-existing Type 2 diabetes mellitus in pregnancy, childbirth and the puerperium	Diabetes
O2411	Pre-existing diabetes mellitus, Type 2, in pregnancy, non-insulin treated	Diabetes
O2412	Pre-existing Type 2 diabetes mellitus in pregnancy, childbirth and the puerperium, insulin treated	Diabetes
O2413	Pre-existing Type 2 diabetes mellitus in pregnancy, childbirth and the puerperium, oral hypoglycaemic therapy	Diabetes
O2414	Pre-existing Type 2 diabetes mellitus in pregnancy, childbirth and the puerperium, other	Diabetes
O2419	Pre-existing Type 2 diabetes mellitus in pregnancy, childbirth and the puerperium, unspecified	Diabetes
O2422	Pre-existing other specified diabetes mellitus in pregnancy, childbirth and the puerperium, insulin treated	Diabetes
O243	Pre-existing unspecified diabetes mellitus, in pregnancy, childbirth and the puerperium	Diabetes
O2431	Pre-existing diabetes mellitus, unspecified, in pregnancy, non-insulin treated	Diabetes

O2432	Pre-existing unspecified diabetes mellitus in pregnancy, childbirth and the puerperium, insulin treated	Diabetes
O2433	Pre-existing unspecified diabetes mellitus in pregnancy, childbirth and the puerperium, oral hypoglycaemic therapy	Diabetes
O2434	Pre-existing unspecified diabetes mellitus in pregnancy, childbirth and the puerperium, other	Diabetes
O2439	Pre-existing unspecified diabetes mellitus in pregnancy, childbirth and the puerperium, unspecified	Diabetes
O244	Diabetes mellitus arising during pregnancy	Diabetes
O2441	Diabetes mellitus arising at or after 24 weeks gestation, non-insulin treated	Diabetes
O2442	Diabetes mellitus arising during pregnancy, insulin treated	Diabetes
O2443	Diabetes mellitus arising during pregnancy, oral hypoglycaemic therapy	Diabetes
O2444	Diabetes mellitus arising during pregnancy, other	Diabetes
O2449	Diabetes mellitus arising during pregnancy, unspecified	Diabetes
O2499	Diabetes mellitus in pregnancy, childbirth and the puerperium, unspecified onset, unspecified	Diabetes
O25	Malnutrition in pregnancy, childbirth and the puerperium	Obsteric medical
O260	Excessive weight gain in pregnancy	Obsteric medical
O261	Low weight gain in pregnancy	Obsteric medical
O264	Pemphigoid gestationis [herpes gestationis]	Obsteric medical
O266	Liver disorders in pregnancy, childbirth and the puerperium	Obsteric medical
O267	Subluxation of symphysis (pubis) in pregnancy, childbirth and the puerperium	Obsteric medical
O2681	Kidney disorders in pregnancy, childbirth and the puerperium	Obsteric medical
O2682	Carpal tunnel syndrome in pregnancy	Obsteric medical
O2683	Neuralgia in pregnancy	Obsteric medical
O2688	Other specified pregnancy-related conditions	Obsteric medical
O280	Abnormal haematological finding on antenatal screening of mother	Obsteric medical
O281	Abnormal biochemical finding on antenatal screening of mother	Obsteric medical
O283	Abnormal ultrasonic finding on antenatal screening of mother	Obsteric medical
O285	Abnormal chromosomal and genetic finding on antenatal screening of mother	Obsteric medical
O288	Other abnormal findings on antenatal screening of mother	Obsteric medical

O289	Abnormal finding on antenatal screening of mother, unspecified	Obsteric medical
O294	Spinal and epidural anaesthesia-induced headache during pregnancy	Obsteric medical
O311	Continuing pregnancy after abortion of one fetus or more	Obsteric medical
O320	Maternal care for unstable lie	Obsteric medical
O321	Maternal care for breech presentation	Obsteric medical
O322	Maternal care for transverse and oblique lie	Obsteric medical
O323	Maternal care for face, brow and chin presentation	Obsteric medical
O324	Maternal care for high head at term	Obsteric medical
O325	Maternal care for multiple gestation with malpresentation of one fetus or more	Obsteric medical
O326	Maternal care for compound presentation	Obsteric medical
O328	Maternal care for other malpresentation of fetus	Obsteric medical
O329	Maternal care for malpresentation of fetus, unspecified	Obsteric medical
O330	Maternal care for disproportion due to deformity of maternal pelvic bones	Obsteric medical
O331	Maternal care for disproportion due to generally contracted pelvis	Obsteric medical
O333	Maternal care for disproportion due to outlet contraction of pelvis	Pre labour complication
O335	Maternal care for disproportion due to unusually large fetus	LGA
O337	Maternal care for disproportion due to other fetal deformities	Fetal indications
O338	Maternal care for disproportion of other origin	Obsteric medical
O339	Maternal care for disproportion, unspecified	Obsteric medical
O340	Maternal care for congenital malformation of uterus	Obsteric medical
O341	Maternal care for tumour of corpus uteri	Obsteric medical
O342	Maternal care due to uterine scar from previous surgery	Obsteric medical
O343	Maternal care for cervical incompetence	Obsteric medical
O344	Maternal care for other abnormalities of cervix	Obsteric medical
O345	Maternal care for other abnormalities of gravid uterus	Obsteric medical
O346	Maternal care for abnormality of vagina	Obsteric medical
O348	Maternal care for other abnormalities of pelvic organs	Obsteric medical
O350	Maternal care for (suspected) central nervous system malformation in fetus	Fetal indications
O351	Maternal care for (suspected) chromosomal abnormality in fetus	Fetal indications
O352	Maternal care for (suspected) hereditary disease in fetus	Fetal indications
O353	Maternal care for (suspected) damage to fetus from viral disease in mother	Fetal indications
O354	Maternal care for (suspected) damage to fetus from alcohol	Fetal indications

O355	Maternal care for (suspected) damage to fetus by drugs	Fetal indications
O357	Maternal care for (suspected) damage to fetus by other medical procedures	Fetal indications
O358	Maternal care for other (suspected) fetal abnormality and damage	Fetal indications
O359	Maternal care for (suspected) fetal abnormality and damage, unspecified	Fetal indications
O360	Maternal care for rhesus isoimmunisation	Fetal indications
O361	Maternal care for other isoimmunisation	Fetal indications
O362	Maternal care for hydrops fetalis	Fetal indications
O363	Maternal care for signs of fetal hypoxia	Fetal indications
O365	Maternal care for poor fetal growth	SGA
O366	Maternal care for excessive fetal growth	LGA
O368	Maternal care for other specified fetal problems	Decreased fetal movements
O369	Maternal care for fetal problem, unspecified	Fetal indications
O40	Polyhydramnios	Fetal indications
O410	Oligohydramnios	Fetal indications
O411	Infection of amniotic sac and membranes	Fetal indications
O418	Other specified disorders of amniotic fluid and membranes	Fetal indications
O419	Disorder of amniotic fluid and membranes, unspecified	Fetal indications
O420	Premature rupture of membranes, onset of labour within 24 hours	TPROM
O4211	Premature rupture of membranes, onset of labour between 1-7 days later	TPROM
O4212	Premature rupture of membranes, onset of labour more than 7 days later	TPROM
O422	Premature rupture of membranes, labour delayed by therapy	TPROM
O429	Premature rupture of membranes, unspecified	TPROM
O430	Placental transfusion syndromes	Fetal indications
O431	Malformation of placenta	Fetal indications
O432	Morbidly adherent placenta	Pre labour complication
O438	Other placental disorders	Pre labour complication
O439	Placental disorder, unspecified	Pre labour complication
O440	Placenta praevia specified as without haemorrhage	Pre labour complication
O441	Placenta praevia with haemorrhage	Pre labour complication
O450	Premature separation of placenta with coagulation defect	Pre labour complication
O459	Premature separation of placenta, unspecified	Pre labour complication
O468	Other antepartum haemorrhage	Pre labour complication
O469	Antepartum haemorrhage, unspecified	Pre labour complication

O470	False labour before 37 completed weeks of gestation	Pre labour complication
O471	False labour at or after 37 completed weeks of gestation	Pre labour complication
O479	False labour, unspecified	Pre labour complication
O48	Prolonged pregnancy	Prolonged pregnancy
O60	Preterm labour and delivery	Pre labour complication
O620	Primary inadequate contractions	Pre labour complication
O622	Other uterine inertia	Pre labour complication
O623	Precipitate labour	Pre labour complication
O624	Hypertonic, incoordinate, and prolonged uterine contractions	Pre labour complication
O630	Prolonged first stage (of labour)	Pre labour complication
O631	Prolonged second stage (of labour)	Pre labour complication
O639	Long labour, unspecified	Pre labour complication
O645	Labour and delivery affected by compound presentation	Pre labour complication
O660	Labour and delivery affected by shoulder dystocia	Pre labour complication
O662	Labour and delivery affected by unusually large fetus	LGA
O665	Failed application of vacuum extractor and forceps, unspecified	Pre labour complication
O679	Intrapartum haemorrhage, unspecified	Pre labour complication
O680	Labour and delivery complicated by fetal heart rate anomaly	Fetal indications
O681	Labour and delivery complicated by meconium in amniotic fluid	Fetal indications
O682	Labour and delivery complicated by fetal heart rate anomaly with meconium in amniotic fluid	Fetal indications
O688	Labour and delivery complicated by other evidence of fetal stress	Fetal indications
O689	Labour and delivery complicated by fetal stress, unspecified	Fetal indications
O692	Labour and delivery complicated by other cord entanglement, with compression	Fetal indications
O693	Labour and delivery complicated by short cord	Fetal indications
O695	Labour and delivery complicated by vascular lesion of cord	Fetal indications
O702	Third degree perineal laceration during delivery	Pre labour complication
O716	Obstetric damage to pelvic joints and ligaments	Pre labour complication
O717	Obstetric haematoma of pelvis	Pre labour complication
O718	Other specified obstetric trauma	Pre labour complication
O7182	Diastasis of recti abdominal muscle in pregnancy or delivery	Pre labour complication
O740	Aspiration pneumonitis due to anaesthesia during labour and delivery	Pre labour complication

O748	Other complications of anaesthesia during labour and delivery	Pre labour complication
O750	Maternal distress during labour and delivery	Pre labour complication
O752	Pyrexia during labour, not elsewhere classified	Pre labour complication
O755	Delayed delivery after artificial rupture of membranes	Pre labour complication
O756	Delayed delivery after spontaneous or unspecified rupture of membranes	Pre labour complication
O757	Vaginal delivery following previous caesarean section	Pre labour complication
O758	Other specified complications of labour and delivery	Pre labour complication
O82	Single delivery by caesarean section	Pre labour complication
O871	Deep phlebothrombosis in the puerperium	Obsteric medical
O878	Other venous complications in the puerperium	Obsteric medical
O880	Obstetric air embolism	Obsteric medical
O882	Obstetric blood clot embolism	Obsteric medical
O888	Other obstetric embolism	Obsteric medical
O903	Cardiomyopathy in the puerperium	Obsteric medical
O9221	Cracked nipple associated with childbirth, with mention of attachment difficulty	Obsteric medical
O982	Gonorrhoea in pregnancy, childbirth and the puerperium	Obsteric medical
O984	Viral hepatitis in pregnancy, childbirth and the puerperium	Obsteric medical
O989	Unspecified maternal infectious or parasitic disease in pregnancy, childbirth and the puerperium	Obsteric medical
O990	Anaemia in pregnancy, childbirth and the puerperium	Obsteric medical
O9900	Anaemia in pregnancy, childbirth and the puerperium, unspecified	Obsteric medical
O9901	Anaemia in pregnancy	Obsteric medical
O9902	Anaemia in pregnancy, with mention of pre-existing anaemia	Obsteric medical
O991	Other diseases of the blood and blood-forming organs and certain disorders involving the immune mechanism in pregnancy, childbirth and the puerperium	Obsteric medical
O992	Endocrine, nutritional and metabolic diseases in pregnancy, childbirth and the puerperium	Obsteric medical
O993	Mental disorders and diseases of the nervous system in pregnancy, childbirth and the puerperium	Obsteric medical
O9932	Diseases of the nervous system in pregnancy, childbirth and the puerperium	Obsteric medical

O994	Diseases of the circulatory system in pregnancy, childbirth and the puerperium	Obsteric medical
O995	Diseases of the respiratory system in pregnancy, childbirth and the puerperium	Obsteric medical
O996	Diseases of the digestive system in pregnancy, childbirth and the puerperium	Obsteric medical
O997	Diseases of the skin and subcutaneous tissue in pregnancy, childbirth and the puerperium	Obsteric medical
O998	Other specified diseases and conditions in pregnancy, childbirth and the puerperium	Obsteric medical
Q (all codes)	Congenital malformations, deformations and chromosomal abnormalities	Fetal indications
R000	Symptoms, signs and abnormal clinical and laboratory findings, not elsewhere classified	Obsteric medical
R001	Symptoms, signs and abnormal clinical and laboratory findings, not elsewhere classified	Obsteric medical
R002	Symptoms, signs and abnormal clinical and laboratory findings, not elsewhere classified	Obsteric medical
R008	Symptoms, signs and abnormal clinical and laboratory findings, not elsewhere classified	Obsteric medical
R010	Symptoms, signs and abnormal clinical and laboratory findings, not elsewhere classified	Obsteric medical
R011	Symptoms, signs and abnormal clinical and laboratory findings, not elsewhere classified	Obsteric medical
R02	Symptoms, signs and abnormal clinical and laboratory findings, not elsewhere classified	Obsteric medical
R030	Symptoms, signs and abnormal clinical and laboratory findings, not elsewhere classified	Obsteric medical
R040	Symptoms, signs and abnormal clinical and laboratory findings, not elsewhere classified	Obsteric medical
R05	Symptoms, signs and abnormal clinical and laboratory findings, not elsewhere classified	Obsteric medical
R060	Symptoms, signs and abnormal clinical and laboratory findings, not elsewhere classified	Obsteric medical

R068	Symptoms, signs and abnormal clinical and laboratory findings, not elsewhere classified	Obsteric medical
R073	Symptoms, signs and abnormal clinical and laboratory findings, not elsewhere classified	Obsteric medical
R074	Symptoms, signs and abnormal clinical and laboratory findings, not elsewhere classified	Obsteric medical
R098	Symptoms, signs and abnormal clinical and laboratory findings, not elsewhere classified	Obsteric medical
R100	Symptoms, signs and abnormal clinical and laboratory findings, not elsewhere classified	Obsteric medical
R101	Symptoms, signs and abnormal clinical and laboratory findings, not elsewhere classified	Obsteric medical
R102	Symptoms, signs and abnormal clinical and laboratory findings, not elsewhere classified	Obsteric medical
R103	Symptoms, signs and abnormal clinical and laboratory findings, not elsewhere classified	Obsteric medical
R104	Symptoms, signs and abnormal clinical and laboratory findings, not elsewhere classified	Obsteric medical
R11	Symptoms, signs and abnormal clinical and laboratory findings, not elsewhere classified	Obsteric medical
R12	Symptoms, signs and abnormal clinical and laboratory findings, not elsewhere classified	Obsteric medical
R202	Symptoms, signs and abnormal clinical and laboratory findings, not elsewhere classified	Obsteric medical
R208	Symptoms, signs and abnormal clinical and laboratory findings, not elsewhere classified	Obsteric medical
R21	Symptoms, signs and abnormal clinical and laboratory findings, not elsewhere classified	Obsteric medical
R227	Symptoms, signs and abnormal clinical and laboratory findings, not elsewhere classified	Obsteric medical
R252	Symptoms, signs and abnormal clinical and laboratory findings, not elsewhere classified	Obsteric medical

R262	Symptoms, signs and abnormal clinical and laboratory findings, not elsewhere classified	Obsteric medical
R2989	Symptoms, signs and abnormal clinical and laboratory findings, not elsewhere classified	Obsteric medical
R31	Symptoms, signs and abnormal clinical and laboratory findings, not elsewhere classified	Obsteric medical
R32	Symptoms, signs and abnormal clinical and laboratory findings, not elsewhere classified	Obsteric medical
R33	Symptoms, signs and abnormal clinical and laboratory findings, not elsewhere classified	Obsteric medical
R34	Symptoms, signs and abnormal clinical and laboratory findings, not elsewhere classified	Obsteric medical
R400	Symptoms, signs and abnormal clinical and laboratory findings, not elsewhere classified	Obsteric medical
R42	Symptoms, signs and abnormal clinical and laboratory findings, not elsewhere classified	Obsteric medical
R454	Symptoms, signs and abnormal clinical and laboratory findings, not elsewhere classified	Obsteric medical
R456	Symptoms, signs and abnormal clinical and laboratory findings, not elsewhere classified	Obsteric medical
R4581	Symptoms, signs and abnormal clinical and laboratory findings, not elsewhere classified	Obsteric medical
R500	Symptoms, signs and abnormal clinical and laboratory findings, not elsewhere classified	Obsteric medical
R509	Symptoms, signs and abnormal clinical and laboratory findings, not elsewhere classified	Obsteric medical
R51	Symptoms, signs and abnormal clinical and laboratory findings, not elsewhere classified	Obsteric medical
R522	Symptoms, signs and abnormal clinical and laboratory findings, not elsewhere classified	Obsteric medical
R529	Symptoms, signs and abnormal clinical and laboratory findings, not elsewhere classified	Obsteric medical

R53	Symptoms, signs and abnormal clinical and laboratory findings, not elsewhere classified	Obsteric medical
R55	Symptoms, signs and abnormal clinical and laboratory findings, not elsewhere classified	Obsteric medical
R568	Symptoms, signs and abnormal clinical and laboratory findings, not elsewhere classified	Obsteric medical
R600	Symptoms, signs and abnormal clinical and laboratory findings, not elsewhere classified	Obsteric medical
R609	Symptoms, signs and abnormal clinical and laboratory findings, not elsewhere classified	Obsteric medical
R634	Symptoms, signs and abnormal clinical and laboratory findings, not elsewhere classified	Obsteric medical
R700	Symptoms, signs and abnormal clinical and laboratory findings, not elsewhere classified	Obsteric medical
R739	Symptoms, signs and abnormal clinical and laboratory findings, not elsewhere classified	Obsteric medical
R748	Symptoms, signs and abnormal clinical and laboratory findings, not elsewhere classified	Obsteric medical
R778	Symptoms, signs and abnormal clinical and laboratory findings, not elsewhere classified	Obsteric medical
R779	Symptoms, signs and abnormal clinical and laboratory findings, not elsewhere classified	Obsteric medical
R790	Symptoms, signs and abnormal clinical and laboratory findings, not elsewhere classified	Obsteric medical
R798	Symptoms, signs and abnormal clinical and laboratory findings, not elsewhere classified	Obsteric medical
R80	Symptoms, signs and abnormal clinical and laboratory findings, not elsewhere classified	Obsteric medical
R81	Symptoms, signs and abnormal clinical and laboratory findings, not elsewhere classified	Obsteric medical
R829	Symptoms, signs and abnormal clinical and laboratory findings, not elsewhere classified	Obsteric medical

R899	Symptoms, signs and abnormal clinical and laboratory findings, not elsewhere classified	Obsteric medical
R943	Symptoms, signs and abnormal clinical and laboratory findings, not elsewhere classified	Obsteric medical
R944	Symptoms, signs and abnormal clinical and laboratory findings, not elsewhere classified	Obsteric medical
R945	Symptoms, signs and abnormal clinical and laboratory findings, not elsewhere classified	Obsteric medical
S (all codes)	Injury, poisoning and certain other consequences of external causes	Non obstetric medical
T (all codes)	Injury, poisoning and certain other consequences of external causes	Non obstetric medical
U91	Syndrome not elsewhere classified	Non obstetric medical
Z028	Other examinations for administrative purposes	Non obstetric medical
Z035	Observation for other suspected cardiovascular diseases	Non obstetric medical
Z038	Observation for other suspected diseases and conditions	Non obstetric medical
Z040	Blood-alcohol and blood-drug test	Non obstetric medical
Z043	Examination and observation following other accident	Non obstetric medical
Z048	Examination and observation for other specified reasons	Non obstetric medical
Z0632	Agent resistant to penicillin and related antibiotics, Methicillin resistant agent	Non obstetric medical
Z208	Contact with and exposure to other communicable diseases	Non obstetric medical
Z209	Contact with and exposure to unspecified communicable disease	Non obstetric medical
Z223	Carrier of other specified bacterial diseases	Non obstetric medical
Z2251	Carrier of viral hepatitis B	Non obstetric medical
Z2252	Carrier of viral hepatitis C	Non obstetric medical
Z238	Need for immunisation against other single bacterial diseases	Non obstetric medical
Z240	Need for immunisation against poliomyelitis	Non obstetric medical
Z268	Need for immunisation against other specified single infectious diseases	Non obstetric medical
Z290	Isolation	Non obstetric medical
Z292	Prophylactic pharmacotherapy	Non obstetric medical
Z312	In vitro fertilisation	Obsteric medical
Z340	Supervision of normal first pregnancy	Elective
Z348 2	Supervision of other normal pregnancy	Elective
Z349	Supervision of normal pregnancy, unspecified	Elective

Z350	Supervision of pregnancy with history of infertility	Elective
Z351	Supervision of pregnancy with history of abortive outcome	Obsteric medical
Z352	Supervsn preg w oth poor obst history	Obsteric medical
Z353	Supervsn preg w h/o insuff A/N care	Obsteric medical
Z354	Supervsn preg w grand multiparity	Obsteric medical
Z355	Supervsn of preg w advanced mat age	Advanced maternal age
Z3551	Supervision of primigravida with advanced maternal age	Advanced maternal age
Z3552	Supervision of multigravida with advanced maternal age	Advanced maternal age
Z356	Supervision of (very) young primigravida	Obsteric medical
Z358	Supervision of other high-risk pregnancies	Obsteric medical
Z359	Supervision of high-risk pregnancy, unspecified	Obsteric medical
Z362	Other antenatal screening based on amniocentesis	Obsteric medical
Z368	Other antenatal screening	Obsteric medical
Z385	Twin, unspecified as to place of birth	Pre labour complication
Z392	Routine postpartum follow-up	Obsteric medical
Z428	Follow-up care involving plastic surgery of other body part	Non obstetric medical
Z438	Attention to other artificial openings	Non obstetric medical
Z466	Fitting and adjustment of urinary device	Non obstetric medical
Z480	Attention to surgical dressings and sutures	Non obstetric medical
Z5188	Other specified medical care	Non obstetric medical
Z5200	Whole blood donor	Non obstetric medical
Z532	Procedure not carried out because of patient's decision for other and unspecified reasons	Non obstetric medical
Z588	Other problems related to education and literacy	Psychosocial
Z591	Inadequate housing	Psychosocial
Z598	Other problems related to housing and economic circumstances	Psychosocial
Z634	Disappearance and death of family member	Psychosocial
Z6379	Other stressful life events affecting family and household	Psychosocial
Z638	Other specified problems related to primary support group	Psychosocial
Z641	Problems related to multiparity	Psychosocial
Z651	Imprisonment and other incarceration	Psychosocial
Z653	Problems related to other legal circumstances	Psychosocial
Z720	Tobacco use, current	Psychosocial

Z722	Drug use	Psychosocial
Z735	Social role conflict, not elsewhere classified	Psychosocial
Z739	Problem related to life-management difficulty, unspecified	Psychosocial
Z740	Need for assistance due to reduced mobility	Psychosocial
Z748	Other problems related to care-provider dependency	Psychosocial
Z752	Other waiting period for investigation and treatment	Psychosocial
Z753	Unavailability and inaccessibility of healthcare facilities	Psychosocial
Z758	Other problems related to medical facilities and other health care	Psychosocial
Z768	Persons encountering health services in other specified circumstances	Psychosocial
Z824	Family history of ischaemic heart disease and other diseases of the circulatory system	Non obstetric medical
Z827	Family history of congenital malformations, deformations and chromosomal abnormalities	Non obstetric medical
Z832	Family history of diseases of the blood and blood-forming organs and certain disorders involving the immune mechanism	Non obstetric medical
Z848	Family history of other specified conditions	Non obstetric medical
Z850	Personal history of malignant neoplasm of digestive organs	Non obstetric medical
Z852	Personal history of malignant neoplasm of other respiratory and intrathoracic organs	Non obstetric medical
Z857	Personal history of other malignant neoplasms of lymphoid, haematopoietic and related tissues	Non obstetric medical
Z858	Personal history of malignant neoplasms of other organs and systems	Non obstetric medical
Z860	Personal history of other neoplasms	Non obstetric medical
Z8610	Personal history of unspecified infectious and parasitic disease	Non obstetric medical
Z8618	Personal history of other infectious and parasitic disease	Non obstetric medical
Z862	Personal history of diseases of the blood and blood-forming organs and certain disorders involving the immune mechanism	Non obstetric medical
Z863	Personal history of endocrine, nutritional and metabolic diseases	Non obstetric medical
Z8642	Personal history of drug use disorder	Non obstetric medical
Z8643	Personal history of tobacco use disorder	Non obstetric medical
Z865	Personal history of other mental and behavioural disorders	Non obstetric medical
Z866	Personal history of diseases of the nervous system and sense organs	Non obstetric medical

Z8669	Personal history of diseases of the nervous system and sense organs, not elsewhere classified	Non obstetric medical
Z867	Personal history of diseases of the circulatory system	Non obstetric medical
Z8672	Personal history of thrombosis and embolism	Non obstetric medical
Z8679	Personal history of diseases of the circulatory system, not elsewhere classified	Non obstetric medical
Z870	Personal history of diseases of the respiratory system	Non obstetric medical
Z8710	Personal history of unspecified digestive disease	Non obstetric medical
Z8718	Personal history of other digestive system disease	Non obstetric medical
Z872	Personal history of diseases of the skin and subcutaneous tissue	Non obstetric medical
Z873	Personal history of diseases of the musculoskeletal system and connective tissue	Non obstetric medical
Z874	Personal history of diseases of the genitourinary system	Non obstetric medical
Z875	Personal history of complications of pregnancy, childbirth and the puerperium	Non obstetric medical
Z876	Personal history of certain conditions arising in the perinatal period	Non obstetric medical
Z877	Personal history of congenital malformations, deformations and chromosomal abnormalities	Non obstetric medical
Z878	Personal history of other specified conditions	Non obstetric medical
Z901	Acquired absence of breast(s)	Non obstetric medical
Z915	Personal history of self-harm	Non obstetric medical
Z916	Personal history of other physical trauma	Non obstetric medical
Z918	Personal history of other specified risk-factors, not elsewhere classified	Non obstetric medical
Z920	Personal history of contraception	Non obstetric medical
Z921	Personal history of long term (current) use of anticoagulants	Non obstetric medical
Z9228	Personal history of long-term [current] use of other medicaments	Non obstetric medical
Z924	Personal history of major surgery, not elsewhere classified	Non obstetric medical
Z928	Personal history of other medical treatment	Non obstetric medical
Z940	Kidney transplant status	Non obstetric medical
Z945	Skin transplant status	Non obstetric medical
Z950	Presence of cardiac device	Non obstetric medical
Z952	Presence of prosthetic heart valve	Non obstetric medical
Z960	Presence of urogenital implants	Non obstetric medical

Z9664	Presence of hip implant	Non obstetric medical
Z978	Presence of other specified devices	Non obstetric medical
Z981	Arthrodesis status	Non obstetric medical
