# Supplementary material for: Trends in induction of labour and associated co-morbidities and demographics in Queensland, Australia from 2001 to 2020: a population-based study
Source: BMC Pregnancy Childbirth. 2025 Mar 26;25:354. doi: 10.1186/s12884-025-07379-5 (PMC11938751; doi:10.1186/s12884-025-07379-5)
Supplement: Supplementary file 2 — Supplementary Material 2 [file 12884_2025_7379_MOESM2_ESM.pdf]

Table 1: Percent differences in participant demographics for all women 2001-2020

	2001-2002	2003-2004	2005-2006	2007-2008	2009-2010	2011-2012	2013-2014	2015-2016	2017-2018	2019-2020
	N=73,931	N=73,680	N=80,003	N=87,002	N=88,289	N=89,101	N=89,860	N=87,644	N=84,235	N=82,320
**Maternal age**										
<19	0.0	-0.3%	-0.8%	-0.7%	-0.9%	-1.3%	-1.9%	-3.0%	-3.6%	-4.0%
20-29	0.0	-2.0%	-2.3%	-1.9%	-1.4%	-1.1%	-1.9%	-2.8%	-3.7%	-4.9%
30-34	0.0	1.2%	0.9%	-0.6%	-1.2%	-0.6%	1.2%	2.8%	2.9%	3.9%
35+	0.0	1.1%	2.2%	3.3%	3.4%	3.0%	2.7%	3.0%	4.3%	5.0%
**Gestation (weeks)**										
37-38	0.0	1.2%	1.5%	1.7%	2.7%	4.4%	6.1%	9.1%	12.9%	13.9%
39-40	0.0	0.4%	1.0%	0.1%	-0.7%	-1.9%	-2.9%	-4.2%	-5.5%	-5.4%
41+	0.0	-1.6%	-2.4%	-1.8%	-1.9%	-2.5%	-3.2%	-4.9%	-7.4%	-8.5%
**Country of birth**										
Australia	0.0	-0.9%	-1.4%	-3.2%	-6.6%	-8.2%	-9.7%	-11.2%	-12.8%	-12.7%
UK	0.0	-0.1%	-0.3%	-0.2%	-0.1%	-0.2%	-0.4%	-0.2%	-0.2%	-0.2%
India	0.0	0.1%	0.2%	0.5%	1.6%	2.1%	2.6%	3.2%	3.8%	4.1%
China	0.0	-0.1%	0.0%	0.1%	0.5%	0.9%	1.2%	1.5%	1.6%	1.5%
NZ	0.0	0.3%	0.5%	1.1%	1.7%	1.7%	1.8%	1.4%	1.6%	1.3%
Other	0.0	0.8%	1.2%	1.8%	3.0%	3.7%	4.6%	5.4%	6.1%	6.1%
**Region**	0.0	0.0%	0.0%	0.0%	0.0%	0.0%	0.0%	0.0%	0.0%	0.0%
Major city	0.0	1.4%	1.6%	2.0%	2.8%	3.0%	3.2%	4.7%	6.0%	5.9%
Inner regional	0.0	-0.5%	-0.2%	-0.3%	-0.6%	-0.3%	-0.5%	-1.2%	-1.9%	-1.6%
Outer regional/ remote	0.0	-0.8%	-1.4%	-1.7%	-2.2%	-2.7%	-2.7%	-3.5%	-4.1%	-4.3%
**Indigenous status** (n=835,916)	0.0	0.1%	0.0%	0.0%	0.1%	0.6%	0.7%	1.2%	1.4%	2.0%
**Model of Care** (n=833,656)										
Public hospital	0.0	3.2%	6.1%	17.1%	18.4%	14.6%	10.5%	8.6%	9.9%	8.1%
GP shared care	0.0	-1.7%	-3.0%	-13.3%	-13.9%	-9.2%	-4.6%	-0.8%	0.1%	3.7%
Private Obstetrician	0.0	-1.2%	-2.7%	-3.5%	-4.2%	-5.3%	-6.4%	-8.9%	-10.9%	-12.6%
Private Midwife	0.0	-0.3%	-0.4%	-0.3%	-0.2%	-0.2%	0.6%	1.1%	0.9%	0.8%

Table 2: Percent differences in co-morbidities for all women 2001 - 2020

	2001-2002	2003-2004	2005-2006	2007-2008	2009-2010	2011-2012	2013-2014	2015-2016	2017-2018	2019-2020
	N=73,931	N=73,680	N=80,003	N=87,002	N=88,289	N=89,101	N=89,860	N=87,644	N=84,235	N=82,320
Hypertension	0.0	-0.6%	-1.4%	-1.9%	-2.3%	-2.7%	-2.9%	-2.5%	-1.8%	-1.8%
Gestational diabetes	0.0	0.5%	0.8%	0.7%	1.2%	2.2%	3.6%	6.7%	8.6%	9.0%
Liver disease	0.0	0.0%	0.3%	0.4%	0.3%	0.4%	0.6%	1.1%	1.1%	0.8%
Term premature rupture of	0.0	-0.1%	0.1%	0.6%	1.0%	1.1%	1.3%	1.5%	1.4%	1.5%
Anaemia	0.0	-0.5%	-0.6%	-0.4%	-0.2%	-0.4%	-0.4%	0.6%	4.4%	5.8%
Mental health	0.0	-0.7%	-0.8%	-0.4%	0.0%	0.5%	1.4%	2.9%	5.1%	7.5%
Antepartum haemorrhage	0.0	-0.5%	-0.7%	-0.9%	-0.7%	-0.8%	-0.7%	-0.6%	-0.3%	-0.3%
Prolonged pregnancy	0.0	-0.6%	-0.8%	-1.4%	-3.4%	-4.5%	-5.6%	-6.4%	-7.3%	-7.6%

Table 3: Percent differences in indications for induction of labour for all women 2001 - 2020

	2001-2002	2003-2004	2005-2006	2007-2008	2009-2010	2011-2012	2013-2014	2015-2016	2017-2018	2019-2020
	N=23230	N=22494	N=23910	N=25259	N=25212	N=26434	N=28132	N=30967	N=34912	N=35951
Prolonged pregnancy	0.0	0.4%	0.4%	-0.7%	-7.1%	-13.5%	-17.5%	-22.9%	-28.0%	-29.4%
Diabetes	0.0	0.4%	1.6%	2.9%	3.4%	5.0%	7.0%	10.1%	11.1%	10.9%
Reduced fetal movements	0.0	0.1%	0.0%	-0.4%	-0.5%	0.1%	1.1%	2.9%	7.7%	8.6%
Hypertension	0.0	-0.6%	-1.8%	-2.4%	-2.8%	-3.1%	-3.7%	-4.2%	-4.8%	-5.4%
TPROM	0.0	-0.1%	-1.1%	0.2%	0.6%	-0.2%	-0.5%	-1.7%	-3.3%	-3.8%
Large for gestational age	0.0	0.0%	0.2%	0.3%	0.3%	0.7%	1.0%	2.9%	6.0%	7.7%
Small for gestational age	0.0	-0.2%	-0.4%	-0.7%	0.1%	0.5%	1.3%	2.5%	3.2%	2.8%
Non obstetric medical	0.0	-0.1%	-0.1%	0.0%	0.6%	0.0%	0.3%	0.2%	0.9%	0.8%
Obstetric medical	0.0	0.4%	0.7%	0.7%	0.4%	1.0%	1.2%	1.4%	1.1%	0.8%
Labour complication	0.0	-1.3%	-1.5%	-1.4%	0.3%	-0.7%	-0.5%	-0.3%	-0.2%	-0.4%
Fetal indication	0.0	-0.4%	-0.3%	-0.1%	0.2%	0.9%	1.7%	1.8%	1.4%	1.4%
Advanced maternal age	0.0	-0.1%	0.0%	0.1%	0.1%	0.2%	1.2%	2.6%	3.2%	3.0%
Elective	0.0	1.6%	2.3%	1.2%	4.0%	8.7%	7.1%	4.5%	1.7%	3.2%
Psychosocial	0.0	0.1%	0.1%	0.2%	0.1%	0.1%	0.0%	-0.1%	0.0%	0.0%

Table 4: Percent differences in clinical outcomes for nulliparous births 2001 - 2020

	**2001-2002**	**2003-2004**	**2005-2006**	**2007-2008**	**2009-2010**	**2011-2012**	**2013-2014**	**2015-2016**	**2017-2018**	**2019-2020**
	N=32,462	N=31,773	N=34,187	N=37,564	N=38,917	N=39,703	N=40,326	N=38,766	N=37,111	N=37,085
IOL	0.0	-0.6%	-0.9%	-1.6%	-2.5%	-1.1%	0.9%	5.0%	11.0%	13.0%
Spontaneous onset of labour	0.0	0.6%	0.9%	1.6%	2.5%	1.1%	-0.9%	-5.0%	-11.0%	-13.0%
Augmentation (n=325,826)	0.0	0.7%	-1.2%	-0.8%	-3.0%	-4.4%	-4.0%	-1.8%	-0.8%	-3.2%
Epidural analgesia	0.0	1.8%	3.0%	3.9%	4.4%	6.1%	8.3%	10.7%	15.2%	18.0%
Mode of birth	0.0	0.0%	0.0%	0.0%	0.0%	0.0%	0.0%	0.0%	0.0%	0.0%
Unassisted vaginal birth	0.0	-2.2%	-4.1%	-6.4%	-6.9%	-7.8%	-7.9%	-8.6%	-10.6%	-10.6%
Assisted vaginal birth	0.0	0.8%	1.3%	3.1%	4.5%	4.9%	5.9%	6.8%	7.0%	6.6%
Caesarean section	0.0	1.4%	2.8%	3.3%	2.4%	2.9%	2.0%	1.8%	3.6%	4.0%
Perineal status (n=431,645)	0.0	0.0%	0.0%	0.0%	0.0%	0.0%	0.0%	0.0%	0.0%	0.0%
Intact	0.0	-0.9%	-1.7%	-5.7%	-9.0%	-9.3%	-11.1%	-13.2%	-14.5%	-14.6%
1st degree	0.0	-1.4%	-1.5%	0.2%	1.5%	-0.2%	-2.8%	-4.3%	-6.3%	-7.7%
2nd degree	0.0	-0.6%	0.6%	2.3%	3.8%	3.8%	4.6%	4.8%	3.6%	3.8%
3rd/4th degree	0.0	0.0%	0.2%	0.8%	0.9%	1.2%	0.9%	0.4%	0.1%	-0.2%
Episiotomy	0.0	2.7%	2.4%	2.2%	2.7%	4.4%	8.3%	12.2%	16.9%	18.6%
Infant birth weight > 4200g	0.0	-0.3%	-0.2%	-0.1%	-0.4%	-0.3%	-0.9%	-1.6%	-2.3%	-2.1%

Table 5: Percent differences in clinical outcomes for multiparous births 2001 - 2020

	**2001-2002**	**2003-2004**	**2005-2006**	**2007-2008**	**2009-2010**	**2011-2012**	**2013-2014**	**2015-2016**	**2017-2018**	**2019-2020**
	N=41,469	N=41,907	N=45,816	N=49,438	N=49,372	N=49,398	N=49,534	N=48,878	N=47,124	N=45,235
IOL	0.0	-1.0%	-1.9%	-2.9%	-3.1%	-2.3%	-1.0%	3.0%	9.3%	11.5%
Spontaneous onset of labour	0.0	1.0%	1.9%	2.9%	3.1%	2.3%	1.0%	-3.0%	-9.3%	-11.5%
Augmentation (n=325,826)	0.0	-1.3%	-3.6%	-4.7%	-9.5%	-12.3%	-12.5%	-11.2%	-10.9%	-13.4%
Epidural analgesia	0.0	1.1%	1.2%	1.5%	2.6%	3.4%	4.9%	7.3%	10.1%	12.8%
Mode of birth	0.0	0.0%	0.0%	0.0%	0.0%	0.0%	0.0%	0.0%	0.0%	0.0%
Unassisted vaginal birth	0.0	-0.8%	-1.0%	-2.1%	-2.5%	-2.5%	-2.5%	-3.0%	-3.0%	-4.0%
Assisted vaginal birth	0.0	0.0%	0.1%	0.5%	0.8%	1.2%	1.1%	1.2%	1.3%	1.6%
Caesarean section	0.0	0.8%	0.9%	1.6%	1.7%	1.3%	1.4%	1.8%	1.7%	2.4%
Perineal status (n=431,645)	0.0	0.0%	0.0%	0.0%	0.0%	0.0%	0.0%	0.0%	0.0%	0.0%
Intact	0.0	-0.1%	0.7%	-5.2%	-9.1%	-8.9%	-8.4%	-11.5%	-13.9%	-15.9%

1st degree	0.0	-0.2%	-0.8%	3.1%	5.1%	3.8%	0.9%	1.3%	2.6%	2.4%
2nd degree	0.0	0.3%	0.9%	2.8%	4.6%	5.5%	7.4%	9.2%	9.9%	11.4%
3rd degree	0.0	0.0%	-0.1%	0.1%	0.2%	0.4%	0.4%	0.5%	0.4%	0.4%
Episiotomy	0.0	0.1%	-0.6%	-0.8%	-0.7%	-0.6%	-0.1%	0.5%	1.1%	1.7%
Infant birth weight > 4200g	0.0	-0.1%	-0.5%	-0.3%	-0.2%	-0.2%	-0.9%	-1.7%	-2.7%	-2.9%
