# Supplementary material for: Trends in induction of labour and associated co-morbidities and demographics in Queensland, Australia from 2001 to 2020: a population-based study
Source: BMC Pregnancy Childbirth. 2025 Mar 26;25:354. doi: 10.1186/s12884-025-07379-5 (PMC11938751; doi:10.1186/s12884-025-07379-5)
Supplement: Supplementary file 3 — Supplementary Material 3 [file 12884_2025_7379_MOESM3_ESM.pdf]

Additional file 3

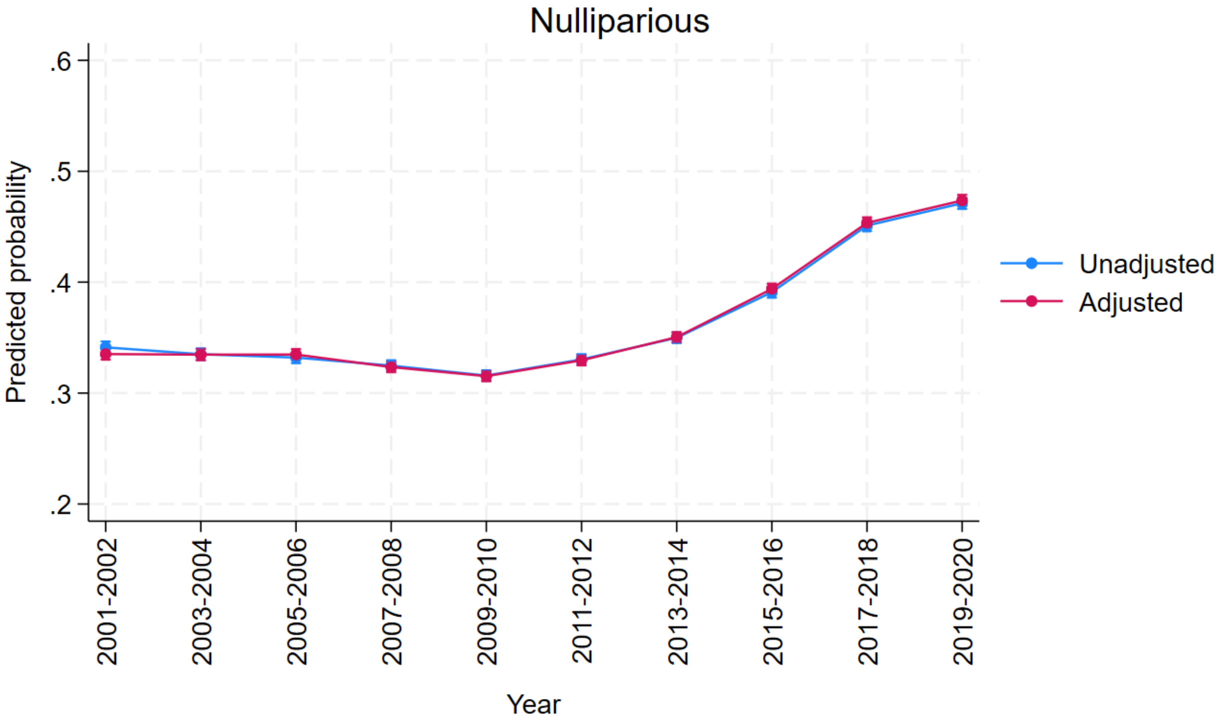


Figure 1: Unadjusted (n=367,894) and adjusted (n=367,348) predicted probability values and 95% CI for IOL and yearly pattern for nulliparous women

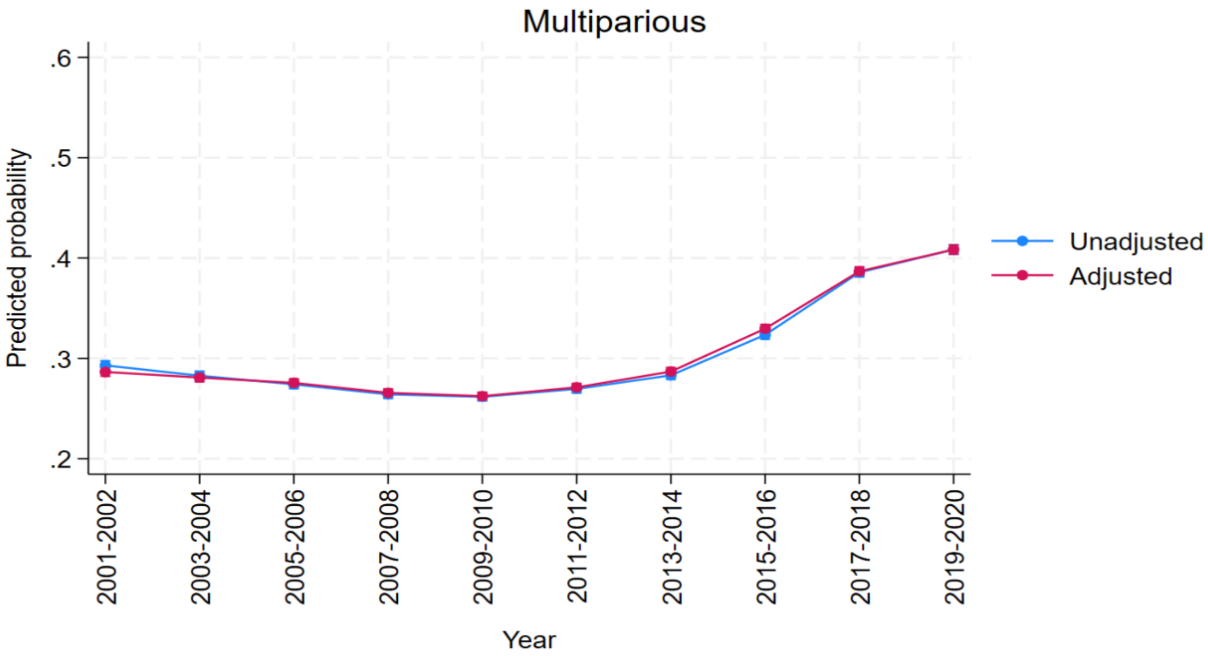


Figure 2: Unadjusted (n=468,171) and adjusted (n=466,308) predicted probability values and 95% CI for IOL and yearly pattern for multiparous women
